# Residue Monitoring and Risk Assessment of 51 Pesticides in Domestic Shellfish and Seaweed Using GC-MS/MS

**DOI:** 10.3390/ijms26104765

**Published:** 2025-05-16

**Authors:** Changkyo Seo, Myungheon Kim, Mihyun Cho, Jaebin Im, Changhyeon Park, Yoonmi Lee, Mi-Ra Jo, Yong-Sun Moon, Moo-Hyeog Im

**Affiliations:** 1 Department of Food Engineering, Daegu University, Gyeongsan 38453, Republic of Korea; 2 Food Safety and Processing Research Division, National Institute Fisheries Science, Busan 46083, Republic of Korea; 3 Department of Horticulture and Life Science, Yeungnam University, Gyeongsan 38541, Republic of Korea

**Keywords:** gas chromatography, monitoring, pesticide, risk assessment, seaweed, shellfish

## Abstract

Many monitoring studies have been performed to assess and manage the risk of residues in seafood contaminated with pesticides owing to various environmental factors. Although seaweed and shellfish have higher consumption rates than fish, studies on their pesticide residues are limited. Therefore, this study aimed to conduct residue monitoring and a risk assessment of 51 pesticides in shellfish (littleneck clam and oyster) and seaweed (sea mustard, seaweed fusiforme, laver, and sea tangle) cultivated in four administrative regions of the Republic of Korea. A total of 120 samples (20 samples per species) were collected, and pesticide residues were analyzed using a modified Quick, Easy, Cheap, Effective, Rugged, and Safe extraction method, followed by a GC-MS/MS analysis. The monitoring results show that oxadiazon was detected at 8–9 ng/g in only four littleneck clam samples. The estimated daily intake was calculated and compared with the acceptable daily intake (ADI) for risk assessment. The %ADI values ranged from 0.05% to 1.12% for average and extreme consumers across six different scenarios. The results of this study suggest that the detected residual levels of pesticides exert no harmful effects on people over a lifetime of consumption.

## 1. Introduction

In modern agriculture, pesticides play a crucial role in increasing crop yields and improving product quality. The development and diversification of pesticides have significantly reduced labor costs for farmers and provided substantial economic benefits. Despite their advantages, pesticides pose risks to human health due to their inherent toxicity, including neurotoxicity, carcinogenicity, and endocrine disruption [1,2,3].

As consumer awareness of pesticide contamination in seafood and its potential health risks has increased recently in Korea, regulatory safety standards have been established and managed for each pesticide to prevent harm due to improper pesticide use [4]. Furthermore, the positive list system, introduced in 2019, mandates a uniform limit of 0.01 mg/kg for pesticides without established residue limits to strengthen the safety of livestock and fishery products [5]. International organizations, such as the Codex Alimentarius Commission (Codex), supported by the Joint FAO/WHO Meeting on Pesticide Residues (JMPR), as well as national regulatory authorities, including the United States Environmental Protection Agency (EPA); the Food and Drug Administration (FDA); the European Commission (EC); and the Ministry of Health, Labour and Welfare (MHLW) in Japan, have established maximum residue limits (MRLs) to ensure the safety of distributed food products, including seafood [6,7].

According to a report from the Korea Rural Economic Institute, seafood consumption in South Korea has been increasing continuously since 2013, driven by rising income levels and attracting attention as a healthy food [8]. As of 2022, the annual per capita consumption amounts of fish, shellfish, and seaweed reached 20.7, 17.0, and 25.6 kg, respectively, summing to a total seafood intake of 63.3 kg, which is comparable to that of the staple food rice (67.4 kg). This finding indicates that seafood is a major part of our diet [9]. Statistical data from the Korea Statistical Office indicate that as of 2023, the total seafood production reached approximately 3.7 million tons, with 62% originating from coastal aquaculture. Among these, seaweed production accounted for 1.76 million tons, shellfish 410,000 tons, and fish 90,000 tons [10,11].

Seaweed and shellfish, which contain proteins, vitamins, and minerals, are widely consumed, owing to their high nutritional value. In particular, seaweed is rich in dietary fiber, iodine, and bioactive compounds, such as fucoidan, while shellfish provide high-quality protein, vitamin B12, taurine, and heme iron. These components are associated with various physiological benefits, including improved liver function, fatigue recovery, antioxidant effects, and enhanced immune response [10,11,12,13]. The littleneck clam (*Protothaca staminea*) is commonly used as a natural flavor enhancer in soups and broths because of its distinctive umami profile, and sea mustard is a highly versatile seaweed ingredient used in various dishes, including soups, stir-fries, and salads [12,13]. However, pesticides used in agriculture can persist in soil and enter aquatic environments through runoff, leading to contamination and accumulation in marine organisms, such as seafood; thus, consumption of contaminated seafood can cause adverse effects on human health [14].

Organochlorine pesticides (OCPs), characterized by high persistence and lipophilicity, are of particular concern because of their ability to accumulate in aquatic environments and organisms. These substances are major pollutants in rivers and lakes and have been classified as “persistent organic pollutants” under the 2001 Stockholm convention [15].

The number of international and domestic studies on pesticide residues in aquatic environments and seafood has increased in recent years. Dirbaba et al. [16] analyzed sediment samples from Ethiopia’s Awash River and detected OCPs, such as heptachlor, heptachlor epoxide, and p,p′-DDT. Nag et al. [17] detected HCH and DDT at 2–540 μg/kg in fish and crustaceans from India’s Chilika Lake. In addition, Park et al. [18] conducted a three-year survey of pesticide residues in the Namhan River, a South Korean watershed, and detected nine pesticides, including carbofuran, at 0.1–22.7 μg/kg. Moreover, Lee et al. [19] monitored pesticide residues in aquatic organisms from six major rivers in South Korea and identified eight pesticide components, including isoprothiolane, at 0.027–12.871 μg/kg.

Shellfish and seaweed are highly susceptible to pesticide contamination from their surrounding environments due to their ability to inhabit diverse ecosystems, low mobility, and long lifespans. In particular, considering the continuously increasing consumption of these seafood products in South Korea, the intake of pesticide-contaminated shellfish and seaweed may pose potential health risks to humans. Nevertheless, studies on pesticide residues in seafood in Korea remain limited. Therefore, this study aimed to investigate the residue levels of pesticides in commonly distributed shellfish and seaweed species in the domestic market and to assess the associated human health risks, thereby providing a scientific basis for ensuring seafood safety.

Therefore, this study aimed to monitor pesticide residues in two shellfish species (littleneck clam and oyster) and four seaweed species (sea mustard, seaweed fusiforme, laver, and sea tangle), with 20 samples collected per species (a total of 120 samples). Gas Chromatography–Tandem Mass Spectrometry (GC-MS/MS) was conducted to detect 51 pesticides, and a risk assessment was performed based on the detected pesticide levels. This study provides essential data for assessing pesticide contamination trends in shellfish and seaweed, as well as developing effective regulatory measures and safety guidelines in South Korea.

## 2. Results and Discussion

### 2.1. Validation of Analytical Method

The limit of detection (LOD) for the 51 target pesticides was determined under a signal-to-noise (S/N) ratio of ≥3 and limit of quantification (LOQ) of ≥10.

The LOD and LOQ were 2–3 and 7–10 ng/g, respectively, depending on the sample. In particular, pesticides with relatively lipophilic properties, such as chlorothalonil, cypermethrin, deltamethrin, hexachlorobenzene, permethrin, prometryn, and tebuconazole [20], had the highest LOQ of 10 ng/g. The coefficient of determination (R^2^) for all the calibration curves was greater than 0.99, confirming a high degree of linearity. Recovery rates were assessed by spiking mixed standard solutions at 1×, 10×, and 50× LOQ into representative species, littleneck clam, and sea mustard. The overall recovery rates and repeatability ranged from 70.1% to 118.5% and 5.13% to 11.40%, respectively, which meet the CODEX guideline criteria of a 70–120% recovery rate and <20% repeatability. The detailed values are summarized in Table 1.

### 2.2. Residue Monitoring Results of Pesticides in Shellfish and Seaweeds

The results of a pesticide residue analysis of the 51 target pesticides are presented in Table 2. Pesticide residues were not detected in any of the 80 seaweed samples tested. Among the 40 shellfish samples, oxadiazon was detected at 8–9 ng/g in four littleneck clam samples. Choi et al. [21] and Seo et al. [22] analyzed OCP residue levels in Manila clam samples collected from the southwestern coast of South Korea, a location highly similar to the sampling area in the present study, and detected DDT. Since the 1970s, DDT has been banned in most Western countries, including South Korea, because of its physiological toxicity and adverse health effects [23]. Although its registration and use are prohibited in South Korea, DDT has a high octanol/water partition coefficient (log Kow 6.2), indicating its high lipophilicity and high bioaccumulation potential. Therefore, DDT may still have some residues in shellfish long after its use has been discontinued [20,24]. Although the types of pesticides detected differ from those in this study, both pesticides share similar physicochemical properties, and further monitoring of pesticide residues is necessary to assess contamination levels in a long-term follow-up survey. Measures should be established to minimize the potential risks to the environment and human health resulting from bioaccumulation. Although no pesticides were detected in the seaweed samples collected in this study, Park et al. [25] detected diuron, an organotoxic antifouling agent used to prevent biofouling of ship hulls, in one laver sample out of 38 seaweed samples, including domestic and imported laver, sea tangle, and sea mustard. Sundhar et al. [26] reported that different seaweed species selectively absorb specific pesticides and that the types and accumulated amounts vary among species. Considering these factors, shellfish and seaweed could be contaminated not only with pesticides but also with various other hazardous substances, which could have long-term impacts. Therefore, continuous monitoring is essential for assessing and managing these potential risks.

Oxadiazon, a selective herbicide detected in littleneck clams, acts as an oxidase inhibitor and is primarily used for pre-emergence weed control in crops such as rice, onions, and potatoes [20,27]. It has a long half-life in soil (3–6 months) and a high octanol/water partition coefficient (log Kow 4.91), indicating its lipophilic nature and strong adsorption potential in biological organisms [20,28]. Kim et al. [29] analyzed 300 aquatic product samples to assess pesticide contamination levels in domestic aquaculture and detected oxadiazon at 7–10 ng/g in crucian carp and far-eastern catfish. They concluded that contamination levels vary depending on the aquaculture environment and pesticide characteristics. Peris et al. [30] also monitored oxadiazon in river water and sediments in the Catalonian region of Spain and found an average oxadiazon concentration of 44 ng/g in sediments and 5.4 ng/L in river water. They suggested that oxadiazon accumulates more in sediment than in water because of its chemical properties. Rodriguez-Gil et al. [31] conducted an agricultural environmental monitoring study as part of Spain’s National Irrigation Plan and detected oxadiazon at 10 ng/g in red swamp crayfish. They inferred that contamination might have occurred either by drainage from irrigation water or by irrigation water itself. Murakami et al. [32] detected oxadiazon at 590–1260 ng/g in mussels collected from the coastal areas of Osaka Bay, Japan. They concluded that oxadiazon undergoes various transformations during metabolic processes and that its contamination level varies depending on its concentration and accumulation degree. Crane et al. [33] analyzed mussels and freshwater clams in California’s agricultural regions and reported oxadiazon concentrations ranging from 16 ng/g to 2400 ng/g on a dry weight basis. They suggested that oxadiazon accumulation in aquatic organisms is likely due to its widespread use in parks, golf courses, and nurseries. Most pesticides, including herbicides, can enter aquatic environments through runoff after use in agriculture and affect the physiological functions of aquatic organisms. Pesticides can be released into water bodies not only through direct application but also during the washing or refilling of spraying equipment [34,35]. Therefore, the detection of oxadiazon in the present study suggests that it may have entered the aquatic environment from the soil and subsequently accumulated in littleneck clams owing to its relatively high lipophilicity. Consequently, systematic management and mitigation measures are necessary to control pesticide use in agricultural areas near aquaculture farms and the surrounding environments.

### 2.3. Risk Assessment of Detected Oxadiazon Pesticide

A risk assessment was conducted for oxadiazon, the only pesticide detected among the 51 monitored pesticide components. Risk was evaluated by calculating the %ADI using the ADI and estimated daily intake (EDI). The consumption of each species is presented in Table 3, and the %ADI results for each scenario, adapted from Kim et al. [29], are summarized in Table 4 and Table 5. Among the six scenarios, Scenario 1, which represents the average consumer by species, resulted in a %ADI of 0.1701%, whereas Scenario 2 yielded 0.1702%. For group-based average consumers, Scenarios 3 and 4 showed very low values of 0.0517% and 0.0522%, respectively. By contrast, for extreme consumers (99th percentile) by species, Scenario 5 resulted in 1.1203%, and Scenario 6 resulted in 1.1233%. According to the common risk assessment guidelines of the Ministry of Food and Drug Safety (MFDS) in South Korea, intentionally used substances such as pesticides pose a risk if the %ADI exceeds 100% [36]. Park et al. [25] monitored oxadiazon residues in 454 fish and 38 seaweed samples from South Korea and performed a risk assessment of catfish and loach. The results show that the average consumer %ADI was 0.046% and 27% for extreme consumers, which was considered safe according to the MFDS criteria. This study noted that determining the precise origin of distributed fish and seaweed is challenging, because they include both domestic and imported products. In addition, washing and cooking are likely to reduce pesticide concentrations, further lowering the actual risk [28]. However, Kim et al. [29] monitored and conducted a risk assessment of farmed aquatic products in South Korea and reported %ADI values for oxadiazon ranging from 0.01% to 1.07% for both average and extreme consumers, indicating no significant potential risk [20]. In the present study, the %ADI of oxadiazon in shellfish and seaweed was <1.12%, showing similar trends. The results confirm that based on both international and domestic standards, consumption of oxadiazon-contaminated shellfish and seaweed presents no potential risks to human health. However, because oxadiazon is a lipophilic pesticide with the potential for bioaccumulation in aquatic organisms, continuous monitoring and risk assessments of other lipophilic pesticides are necessary. Furthermore, this study analyzed only 51 pesticides, which limits its scope. In future studies, the range of pesticide compounds must be expanded, and systematic monitoring along with corresponding risk assessments must be conducted.

## 3. Materials and Methods

### 3.1. Chemicals and Materials

A total of 51 pesticides, including 2,4′-DDD, 2,4′-DDE, 2,4′-DDT, 4,4′-DDD, 4,4′-DDE, 4,4′-DDT, acetochlor, alachlor, aldrin, ametryn, atrazine, boscalid, buprofezin, carfentrazone-ethyl, chlordane-cis, chlordane-trans, chlorothalonil, chlorpyrifos, chlorpyrifos-methyl, cypermethrin, deltamethrin, dieldrin, dimethametryn, diphenylamine, α-endosulfan, β-endosulfan, endosulfan sulfate, endrin, fenitrothion, fipronil, heptachlor, heptachlor epoxide-cis, heptachlor epoxide-trans, hexachlorobenzene, iprobenfos, isoprothiolane, mirex, nonachloer-cis, nonachloer-trans, oxadiazon, pendimethalin, permethrin, prometryn, tebuconazole, terbutryn, tetraconazole, thifluzamide, trifluralin, α-HCH, β-HCH, and γ-HCH, were purchased from Chemidas Co. (Gunpo, Republic of Korea). Analytical-grade solvents for pretreatment were obtained, including acetonitrile (ACN) and hexane from Avantor (Radnor, PA, USA) and dichloromethane from Honeywell (Charlotte, NC, USA). Acetic acid (≥99.7%) and magnesium sulfate (MgSO_4_; 99.5%) were purchased from Sigma-Aldrich (St. Louis, MO, USA), and sodium acetate (NaOAc; 98.5%) was procured from Junsei (Tokyo, Japan). Florisil cartridges (Sep-Pak Florisil 6cc Vac Cartridge; 500 mg; 50–200 μm; 30/pk), used for purification, were purchased from Waters (Wexford, Ireland). Conical tubes (15 and 50 mL) were obtained from SPL (Pocheon, Republic of Korea), and shaking and centrifugation were performed using a Mixer CM-1000 (Rikakikai, Tokyo, Japan) and Megafuge 1.R (Thermo Fisher Scientific, Waltham, MA, USA), respectively.

### 3.2. Sample Collection and Preparation

A total of 120 samples (20 per species) of shellfish (oyster and littleneck clams) and seaweed (sea mustard, seaweed fusiforme, laver, and sea tangle) were collected from aquaculture farms and wholesale markets in four administrative regions of South Korea (Gyeongsangnam-do, Jeollanam-do, Jeollabuk-do, and Chungcheongnam-do). The origin of all species was verified. Shellfish samples were depurated in tap water for 2–3 h, after which the shells and internal organs were removed, retaining only the edible muscle. Seaweed samples were soaked in distilled water for 30 min to remove salt and moisture before homogenization. Each sample (500 g) was homogenized using dry ice in a household blender until the mixture was sufficiently pulverized into a fine powder. Due to variations in sample texture and composition, the blending time was adjusted as needed to ensure adequate homogenization. The homogenized samples were then stored at −20 °C until analysis.

### 3.3. Pesticide Residue Analysis

Pesticide residues were analyzed using a modified version of the method described by Kim et al. [29]. Briefly, 5 g of the homogenized sample was placed in a 50 mL conical tube, followed by the addition of 20 mL of ACN containing 0.1% acetic acid. The mixture was then shaken at 2000 rpm for 20 min. After adding 6 g of MgSO_4_ and 1.5 g of NaOAc, the mixture was shaken for an additional 5 min and centrifuged at 4000× *g* (4 °C) for 10 min. The upper ACN layer (4 mL) was transferred to a 15 mL conical tube containing 0.6 g of MgSO_4_ and centrifuged again. The resulting supernatant (2.5 mL) was evaporated under reduced pressure at 30 °C, and the residue was dissolved in hexane (2.5 mL). Purification was performed using Florisil cartridges preconditioned with 5 mL of hexane (flow rate of 2–3 drops/second). A 2 mL aliquot of the hexane-dissolved extract was loaded onto the cartridge and eluted at the same flow rate. Additional elution with 5 mL of a dichloromethane:acetonitrile:hexane (50.0:3.5:46.5, *v*/*v*/*v*) mixture in 1 mL portions was collected in the same fraction. Each fraction was evaporated to dryness under nitrogen at 40 °C and reconstituted in 1 mL of hexane before GC-MS/MS analysis.

### 3.4. GC-MS/MS Analysis

GC-MS/MS analysis for the qualitative and quantitative determination of the 51 pesticides was performed using an Agilent 7010 B Triple Quad GC/MS with the MassHunter Quantitative Analysis software (Veresion 10.1.49, Agilent Technologies, Santa Clara, CA, USA). The ionization was in the electron ionization mode, and the precursor ions were selected based on the retention time of multiple reaction monitoring by full scan. The most sensitive and selective productions were selected after optimizing collision energies (5–50 eV). The analytical conditions for the GC and MS/MS are summarized in Table 6 and Table S1, respectively.

### 3.5. Validation of Analytical Method

The GC-MS/MS method for the analysis of 51 pesticides was validated using representative shellfish and seaweed samples, namely, littleneck clam and sea mustard, respectively, in accordance with the CODEX CAC/GL-71 guidelines [37]. The method was verified by linearity, LOD, LOQ, recovery, and repeatability tests. Mixed standard solutions were spiked at various concentration levels: LOQ 6 ng/g (1.5, 3, 4.2, 6, 7.2, 9, and 12 ng/g); LOQ 7 ng/g (1.75, 3.5, 4.9, 7, 8.4, 10.5, and 14 ng/g); LOQ 8 ng/g (2, 4, 5.6, 8, 9.6, 12, and 16 ng/g); LOQ 9 ng/g (2.25, 4.5, 6.3, 9, 10.8, 13.5, and 18 ng/g); and LOQ 10 ng/g (2.5, 5, 7, 10, 12, 15, and 20 ng/g). The R^2^ values of the calibration curves were calculated. To minimize errors in the analytical results caused by matrix effects, we used a matrix-matched calibration curve to confirm linearity. The LOD and LOQ were set differently for each pesticide component according to the analytical instrument detection and quantification criteria and were determined using the S/N ratio. Recovery tests were conducted by spiking mixed standard solutions at LOQ, 10× LOQ, and 50× LOQ and performing five replicates. Additionally, the repeatability of the method was assessed by spiking pesticide-free samples at the LOQ level and analyzing them, ensuring that the relative standard deviation remained within 20% as per the guidelines.

### 3.6. Risk Assessment of Pesticide Residues in Shellfish and Seaweed

The risk assessment for the analyzed pesticide components was conducted using %ADI, calculated as the ratio of the EDI to the ADI multiplied by 100. The %ADI is also represented by the health risk index (HI). The EDI was determined by multiplying the detected concentration by the daily food intake and then dividing it by the average body weight of a Korean adult (60 kg bw/person). The calculation methods are shown in Equations (1) and (2). All daily consumption data were obtained from the 2017–2021 National Health and Nutrition Survey, utilizing either average or extreme consumption levels (99th percentile) [38]. For each species, the average and extreme consumption levels were used for littleneck clam, oyster, sea mustard, seaweed fusiforme, laver, and sea tangle. However, extreme consumption data were unavailable for seaweed fusiforme. Thus, half of the extreme consumption value of sea tangle, the lowest among the analyzed seaweed species, was applied. For group-based assessments, only the average consumption of nine shellfish (including littleneck clam and oyster) and four seaweed species (including sea mustard, seaweed fusiforme, laver, and sea tangle) was used.

The HI (%ADI) was calculated based on six different scenarios of daily food intake by group or species, as shown in Table 6. Scenarios 1 and 2 used species-specific average intake levels and extreme intake for Scenarios 5 and 6, whereas group-based average intakes were applied to Scenarios 3 and 4. In Scenarios 1, 3, and 5, the pesticide detection levels in the EDI calculations were determined by summing the concentrations of detected pesticides in each sample and summing all applied LOQ values to non-detected samples. This value was divided by the total number of samples. However, the maximum detected concentration was applied to all detected samples in Scenarios 2, 4, and 6. The remaining calculations were the same.

EDI (mg/kg b.w./day) = Detected pesticide concentration (mg/kg) × Daily food intake (kg/day)/60 kg(1)

HI (%ADI) = EDI (mg/kg·b.w./day)/ADI (mg/kg·b.w./day) × 100(2)

### 3.7. Data Analysis

Data analysis was performed using Microsoft Office Excel 2013 (Microsoft Corporation, Seattle, WA, USA). The mean and standard deviation (SD) values were calculated from five replicates.

## 4. Conclusions

In this study, residue monitoring and a risk assessment of six species of shellfish (littleneck clam and oyster) and seaweed (sea mustard, seaweed fusiforme, laver, and sea tangle) were conducted in four administrative regions in South Korea. The herbicide oxadiazon was detected in four littleneck clam samples. This contamination may be attributed to the accumulation of pesticides in agricultural areas near aquaculture farms, which were then transported into the aquatic environment through runoff or erosion. The risk assessment, based on six different scenarios, indicated that the %ADI values were very low, confirming that even in cases of average or extreme consumption, no harmful effects on human health would occur. These findings provide essential data for ensuring the safety of seafood products and contribute to the development of a reliable and healthy food culture for consumers. However, the actual risk may vary, depending on individual consumption patterns and preferences. Additionally, the possibility of contamination from pesticides was not detected in this study; other pesticide compounds were not included in the monitoring, and various environmental factors could not be ruled out. Therefore, continuous and systematic monitoring and further research are necessary to ensure long-term food safety.

## Figures and Tables

**Table 1:** Linearity, limit of detection (LOD), limit of quantitation (LOQ), recovery, and repeatability of 51 multi-class pesticides in two representative species (littleneck clam and sea mustard).

Pesticides	Linearity (R^2^)	LOD (ng/g)	LOQ (ng/g)	Recovery Rate (%)			CV ^a^ (%)
				LOQ	10× LOQ	50× LOQ	
2,4′-DDD	0.9967–0.9998	2	7	98.7–105.7	100.4–108.8	101.1–114	8.06–9.52
2,4′-DDE	0.9972–0.9999	2	7	93.9–99.5	92.0–101.4	94.1–105.8	8.79–9.53
2,4′-DDT	0.9963–0.9997	2	7	101.0–105.9	100.4–109.9	101.9–115.1	8.89–9.73
4,4′-DDD	0.9954–0.9987	2	7	83.1–113.3	74.7–112.2	70.4–111.8	8.97–10.22
4,4′-DDE	0.9939–0.9998	2	7	95.3–101.8	92.1–100.7	94.3–104.9	8.00–9.53
4,4′-DDT	0.9934–0.9994	2	7	76.9–108.3	85.1–109.7	70.5–107.9	6.85–10.05
Acetochlor	0.9927–0.9983	2	7	99.8–100.5	88.1–106.4	88.6–110.4	9.21–11.04
Alachlor	0.9988–0.9999	2	7	84.3–108.6	78.0–104.8	80.6–107.1	9.42–9.58
Aldrin	0.9915–0.9989	2	7	86.9–88.5	87.0–88.8	89.5–93.6	8.46–9.42
Ametryn	0.9944–0.9999	2	7	92.7–106.3	89.2–112.4	78.9–110.1	7.85–8.30
Atrazine	0.9973–0.9982	2	7	90.3–103.2	86.5–105.5	87.7–112.4	8.29–8.55
α-BHC	0.9988–0.9999	2	7	81.6–94.9	81.4–101.1	86.2–106.3	9.00–9.20
β-BHC	0.9987–0.9996	2	7	86.2–90.4	91.7–91.8	94.9–97.1	8.80–9.74
γ-BHC	0.9996–0.9951	2	7	84.5–89.0	83.3–92.2	88.7–97.3	7.05–9.11
Boscalid	0.9950–0.9987	2	7	77.3–109.4	96.5–114.7	79.9–82.1	7.37–10.85
Buprofezin	0.9908–0.9966	2	7	100.2–109.9	92.6–111.0	89.7–114.3	9.75–10.62
Carfentrazone-ethyl	0.9922–0.9990	2	7	90.0–104.1	81.4–102.7	77.9–115.6	8.52–10.35
α-Chlordane	0.9926–0.9994	2	7	95.7–96.0	91.8–99.9	96.1–100.1	8.52–9.50
β-Chlordane	0.9997–0.9980	2	7	91.8–94.2	89.8–100.8	93.8–103.8	9.49–9.79
Chlorothalonil	0.9978–0.9990	2	7	84.3–84.5	81.4–88.8	81.0–89.3	8.61–9.08
Chlorpyrifos	0.9991–0.9993	2	7	89.9–108.1	86.9–105.3	88.5–108.8	8.62–10.96
Chlorpyrifos-methyl	0.9991–0.9998	2	7	87.7–102.3	84.9–102.2	87.2–106.0	8.11–8.59
Cypermethrin	0.9954–0.9995	3	10	80.0–113.0	95.2–115.3	79.0–80.5	8.10–10.98
Deltamethrin	0.9987–0.9988	3	10	83.3–110.6	93.4–118.5	72.0–72.8	9.88–11.40
Dieldrin	0.9988–0.9981	2	7	87.6–99.8	82.2–102.3	82.8–104.1	8.99–9.87
Dimethametryn	0.9983–0.9996	2	7	88.1–111.0	86.6–113.8	83.5–117.9	9.30–10.01
Diphenylamine	0.9987–0.9989	3	7–10	73.2–89.1	72.0–89.2	84.3–95.4	8.94–9.46
Endosulfan sulfate	0.9975–0.9989	2	7	91.2–105.9	88.0–109.5	87.6–114.1	8.17–9.97
α-Endosulfan	0.9991–0.9982	2	7	88.9–108.7	93.0–106.9	90.1–91.7	8.72–9.35
β-Endosulfan	0.9933–0.9987	2	7	90.0–94.8	91.1–93.0	95.0–97.1	9.38–9.88
Endrin	0.9927–0.9995	2	7	93.0–99.6	87.0–103.0	93.1–105.4	8.95–10.07
Fenitrothion	0.9994–0.9998	2	7	88.5–97.6	82.1–105.0	80.6–109.0	8.28–10.14
Fipronil	0.9982–0.9994	2	7	88.7–106.4	79.2–103.1	79.4–107.1	7.59–10.72
Heptachlor	0.9977–0.9994	2	7	81.5–110.5	75.2–113.0	79.3–115.8	8.54–9.45
Heptachlor epoxide (cis)	0.9946–0.9991	2	7	97.0–107.0	95.5–109.9	90.9–100.3	8.63–9.51
Heptachlor epoxide (trans)	0.9992–0.9977	2	7	89.5–93.9	92.8–95.0	93.3–97.4	6.16–9.42
Hexachlorobenzene	0.9987–0.9994	3	7–10	71.6–89.9	70.1–96.1	74.5–97.1	8.62–8.79
Iprobenfos	0.9989–0.9998	2	7	88.3–93.0	82.2–96.4	78.4–100.8	8.10–8.39
Isoprothiolane	0.9996–0.9985	2	7	80.8–94.7	83.4–86.2	80.8–86.2	7.25–9.51
Mirex	0.9998–0.9997	2	7	89.4–110.5	86.9–114.3	90.9–113.9	7.73–9.69
Nonachlor (cis)	0.9970–0.9966	2	7	97.1–97.8	95.8–97.2	96.2–106.0	8.56–8.85
Nonachlor (trans)	0.9995–0.9980	2	7	94.3–96.1	95.1–99.1	97.1–103.9	7.52–8.55
Oxadiazon	0.9979–0.9999	2	7	94.8–97.1	88.4–102.2	89.9–107.7	8.29–8.48
Pendimethalin	0.9930–0.9979	2	7	88.1–99.4	81.5–103.9	81.2–105.7	7.90–9.71
Permethrin	0.9957–0.9962	2	7	85.2–110.6	78.5–109.0	77.2–110.9	7.07–10.83
Prometryn	0.9984–0.9989	2	7	93.1–107.1	91.4–112.9	87.4–115.7	8.49–8.72
Tebuconazole	0.9918–0.9941	3	10	71.6–95.0	70.7–95.8	103.6–111.4	5.13–10.76
Terbutryn	0.9975–0.9998	3	9–10	100.7–109.6	75.0–109.3	73.3–111.5	9.32–10.42
Tetraconazole	0.9962–0.9938	2	7	95.7–102.2	91.2–91.4	92.8–108.3	8.91–8.96
Thifluzamide	0.9958–0.9948	2	7	84.2–88.1	72.6–111.1	80.0–115.7	7.86–10.21
Trifluralin	0.9969–0.9994	2	7	84.7–101.7	82.0–87.8	83.0–99.1	8.23–9.16

^a^ CV (%): coefficient of variation indicates repeatability.

**Table 2:** Residue monitoring results of 51 pesticides from the target fish species.

Group	Species	Sample (N)	Detected Pesticide	Detection Number	Min. ^a^ (ng/g)	Max. ^b^ (ng/g)	Mean (ng/g)
Shellfish	Littleneck clam	20	Oxadiazon	4	8.00	9.00	8.75
	Oyster	20	ND ^c^	-	-	-	-
Seaweed	Sea mustard	20	ND	-	-	-	-
	Seaweed fusiforme	20	ND	-	-	-	-
	Laver	20	ND	-	-	-	-
	Sea tangle	20	ND	-	-	-	-

^a^ Min.: minimum concentration of the detected pesticide. ^b^ Max.: maximum concentration of the detected pesticide. ^c^ ND: not detected.

**Table 3:** Korean food consumption by fish species and seafood groups from KNHANES (2017–2021).

Seafood Species			Food Consumption (g/person/day) in KNHANES ^a^	
			Mean	Extreme (99%ile)
Fish species	Shellfish	Littleneck clam	1.0800	25.8000
		Oyster	0.7200	18.7200
	Seaweed	Sea mustard	9.4057	217.8335
		Seaweed fusiforme	0.0000	5.3268 ^c^
		Laver	41.0100	60.900
		Sea tangle	0.0000	10.6537
Group ^b^	Shellfish (9)		4.3628	-
	Seaweed (4)		10.4857	-

^a^ KNHANES: Korea National Health and Nutrition Examination Survey. ^b^ Group: shellfish (9), including littleneck clam, oyster, spiny top shell, abalone, scallop, mussel, granular ark, orient hard clam, and snail. Seaweed (4), including sea mustard (dried and fresh), seaweed fusiforme, laver (seasoned laver and crispy seaweed flakes), and sea tangle. ^c^ Extreme (99%ile) food consumption of seaweed fusiforme (5.3268) represented half of the sea tangle value (10.6537), because it did not have original data.

**Table 4:** Six scenarios for the risk assessment of oxadiazon.

EDI Scenario ^a^	Oxadiazon Pesticide Concentration for Samples		Daily Food Intake
S1	(Sum of all detected pesticide concentrations + sum of LOQ for non-detected samples) /number of tested samples (20)	×	Average Intake by fish species
S2	Maximum concentration was used for all detected samples (the rest is the same as S1)		
S3	(Sum of all detected pesticide concentrations + sum of LOQ for non-detected samples) /number of tested samples (shellfish, 40; seaweed, 80)	×	Average intake by group
S4	Maximum concentration was used for all detected samples (the rest is the same as S3)		
S5	(Sum of all detected pesticide concentrations + sum of LOQ for non-detected samples) /number of tested samples (20)	×	Extreme intake by fish species
S6	Maximum concentration was used for all detected samples (the rest is the same as S5)		

^a^ EDI scenario: daily food intake × pesticide concentration for samples by condition. S1 and S2: average intake by fish species. S3 and S4: average intake by fish group. S5 and S6: extreme (99%ile) intake by fish species.

**Table 5:** Risk assessment results for oxadiazon in six scenarios.

Detected Pesticide	Scenario	EDI (μg/person/day)	ADI (μg/person/day)	%ADI ^b^
Oxadiazon	1	0.3674	216 ^a^	0.1701
	2	0.3677		0.1702
	3	0.1116		0.0517
	4	0.1127		0.0522
	5	2.4198		1.1203
	6	2.4262		1.1233

^a^ The ADI 216 (μg/person/day) was calculated by 0.0036 mg/kg body weight oxadiazon (by the residue information of CODEX) multiplied by 60 kg (the average weight of a Korean adult). ^b^ %ADI = EDI/ADI × 100.

**Table 6:** GC-MS/MS condition for residue analysis of 51 pesticides.

Parameters	GC Condition		
Column	DB-5MS UI (30 m × 250 µm × 0.25 µm)		
Flow rate	1.2 mL/min		
Injection volume	1 µL		
Injection mode	Split mode (5:1)		
Carrier gas	He		
Injection temp.	260 °C		
Oven temp.	Rate (°C/min)	Value (°C)	Hold time (min)
		60	0.2
	20	180	0
	15	250	3
	20	300	5
	MS/MS condition		
Ion source	EI		
Source temp.	250 °C		
Electron energy	70 eV		

## Data Availability

The data presented in this study are available upon request from the corresponding author, owing to legal restrictions.

## Supplementary Materials

The following supporting information can be downloaded at: https://www.mdpi.com/article/10.3390/ijms26104765/s1.

## Abbreviations

The following abbreviations are used in this manuscript:

ADI	Acceptable daily intake
OCPs	Organochlorine pesticides
GC-MS/MS	Gas Chromatography–Tandem Mass Spectrometry
LOD	Limit of detection
LOQ	Limit of quantification
EDI	Estimated daily intake
MFDS	Ministry of Food and Drug Safety
MgSO_4_	Magnesium sulfate
NaOAc	Sodium acetate
ACN	Acetonitrile
S/N	Signal-to-noise ratio
HI	Health risk index
R^2^	Coefficient of determination
SD	Standard deviation

## References

[1] Do J.A., Lee H.J., Shin Y.U., Choi W.J., Chae G.Y., Kang C.S., Kim W.S. (2010). Monitoring of pesticide residues in domestically distributed agricultural products: A review. J. Korean Soc. Food Sci. Nutr..

[2] Kim S.H., Choi W.J., Baek Y.G., Kim W.S. (2008). Monitoring and safety evaluation of pesticide residues in domestically distributed agricultural products. Korean J. Food Sci. Nutr..

[3] Jang M.R., Moon H.K., Kim T.R., Yuk D.H., Kim J.H., Park S.K. (2010). Risk assessment of pesticide residues from the consumption of vegetables distributed in the Seoul area. Korean J. Nutr..

[4] Kim J.H., Lee D.B., Lee M.G., Ryu G.Y., Kim T.S., Kang K.R., Kim J.B. (2019). Monitoring and risk assessment of pesticide residues in agricultural products for school meals in Gwangju metropolitan city. J. Food Hyg. S. Afr..

[5] MFDS (2022). https://impfood.mfds.go.kr/CFBBB01F02/getCntntsDetail?cntntsSn=278787&cntntsMngId=00032.

[6] Im M.H., Ji Y.J. (2016). A review on processing factors of pesticide residues during fruits processing. J. Appl. Biol. Chem..

[7] Kim M., Kim T.H., Park J.W., Lee Y., Jo M.R., Moon Y.S., Im M.H. (2024). A robust method for simultaneous determination and risk assessment of multiresidual pesticides in fishery products. Toxics.

[8] MOF (Ministry of Oceans and Fisheries) (2022). Seafood Consumption (Per Person Per Year) and Self-Sufficiency. https://www.index.go.kr/unity/potal/main/EachDtlPageDetail.do?idx_cd=1317.

[9] Lee H.D., Baek E.Y. (2024). Diagnosis of seafood consumption in Korea: Issues in supply-demand estimation and improvement tasks. Korean Isl. Stud..

[10] Shin S.B., Choi W.S., Lee J.H., Kim M.J., Lim C.W. (2022). Assessment of bacteriological safety of the seawater and abalone (Haliotis discus hannai) in the Bogil-Nohwa area, Korea. Korean J. Malacol..

[11] KOSIS (2022). https://kosis.kr/statHtml/statHtml.do?orgId=723&tblId=DT_72301_E033&vw_cd=&list_id=&seqNo=&lang_mode=ko&language=kor&obj_var_id=&itm_id=&conn_path=C1.

[12] Moon J.H., Kim J.T., Kang S.T., Heo J.H., Oh K.S. (2003). Processing and quality characteristics of flavoring materials using short-necked clams (*Ruditapes philippinarum*). Korean J. Fish. Sci. Technol..

[13] Yun S.J. (2007). History of utilization and cooking methods of seaweeds in Korea. Proceedings of the East Asian Society of Dietary Life (EASDL) Conference, Dankook University.

[14] Chang G.R. (2018). Persistent organochlorine pesticides in aquatic environments and fishes in Taiwan and their risk assessment. Environ. Sci. Pollut. Res. Int..

[15] Li Q., Luo Z., Yan C., Zhang X. (2011). Assessment of polychlorinated biphenyls contamination in sediment and organism from Xiamen offshore area, China. Bull. Environ. Contam. Toxicol..

[16] Dirbaba N.B., Li S., Wu H., Yan X., Wang J. (2018). Organochlorine pesticides, polybrominated diphenyl ethers and polychlorinated biphenyls in surficial sediments of the Awash River Basin, Ethiopia. PLoS ONE.

[17] Nag S.K., Saha K., Bandopadhyay S., Ghosh A., Mukherjee M., Raut A., Mohanty S.K. (2020). Status of pesticide residues in water, sediment, and fishes of Chilika Lake, India. Environ. Monit. Assess..

[18] Park K.H., Kim C.S., Park B.J., Lee B.M., Choi J.H., Jeong M.H., Park H.J. (2007). Investigation of pesticide residues in the Bokpo Stream watershed, a tributary of the Namhan River. Korean J. Pestic. Sci..

[19] Lee J.H., Park B.J., Kim J.K., Kim W.I., Hong S.M., Lim G.J., Hong M.G. (2011). Risk assessment of pesticides detected in major river water systems on aquatic organisms. Korean J. Pestic. Sci..

[20] Turner J.A. (2015). The Pesticide Manual: A World Compendium.

[21] Choi J.Y., Yang D.B., Hong G.H., Shin K.H. (2014). Distribution and bioaccumulation of polychlorinated biphenyls and organochlorine pesticides residues in sediments and Manila clams (*Ruditapes philippinarum*) from along the Mid-Western coast of Korea. Mar. Pollut. Bull..

[22] Seo Y.T., Lim G.J., Shim J.H. (1986). Residual evaluation of organochlorine pesticides in sediments of coastal shellfish habitats. Korean J. Environ. Agric..

[23] Thuy T.T. (2015). Effects of DDT on environment and human health. J. Educ. Soc. Sci..

[24] Noh H.J., Yoon S.M., Kim J.I., Yoon J.G., Hwang J.A., Kim I.J., Kim H.G. (2019). Survey on organochlorine pesticides in agricultural soils. Proc. Korean Soc. Environ. Agric..

[25] Park D., Kim J.Y., Jang G.H., Eom M. (2024). Nationwide survey in Korea for risk assessment of pesticide residues in fishery and seaweed products. J. Hazard. Mater..

[26] Sundhar S., Jeyashakila R., Aanand S., Shalini R. (2021). Assessment of organochlorine pesticides in different seaweed species of Thoothukudi coast, Tamil Nadu, South India. Indian J. Fish..

[27] Rahman M.M., Song K.S., Rhee I.K., Kim J.E. (2005). Impact of herbicide oxadiazon on microbial activity and nitrogen dynamics in soil environment. J. Appl. Biol. Chem..

[28] Milan M., Ferrero A., Fogliatto S., Piano S., Negre M., Vidotto F. (2019). Oxadiazon dissipation in water and topsoil in flooded and dry-seeded rice fields. Agronomy.

[29] Kim M., Cho M., Kim S.H., Lee Y., Jo M.R., Moon Y.S., Im M.H. (2024). Monitoring and risk assessment of pesticide residues in fishery products using GC–MS/MS in South Korea. Toxics.

[30] Peris A., Eljarrat E. (2021). Multi-residue methodologies for the analysis of non-polar pesticides in water and sediment matrices by GC–MS/MS. Chromatographia.

[31] Rodriguez-Gil J.L., San Sebastián Sauto J., González-Alonso S., Sánchez Sanchez P., Valcarcel Y., Catalá M. (2013). Development of cost-effective strategies for environmental monitoring of irrigated areas in Mediterranean regions: Traditional and new approaches in a changing world. Agric. Ecosyst. Environ..

[32] Murakami Y., Nishimune T., Sueki K. (1993). Studies on a rice plant pesticide—Oxadiazon and its metabolites in shellfishes. Toxicol. Environ. Chem..

[33] Crane D.B., Younghans-Haug C. (1992). Oxadiazon residue concentrations in sediment, fish, and shellfish from a combined residential/agricultural area in southern California. Bull. Environ. Contam. Toxicol..

[34] Oluah N.S., Mgbenka B.O., Nwani C.D., Aguzie I.O., Ngene I.C., Oluah C. (2020). Tissue-specific changes in Ca^2+^-ATPase and Na^+^/K^+^-ATPase activities in freshwater African catfish Clarias gariepinus juvenile exposed to oxadiazon. J. Basic Appl. Zool. Vol..

[35] Warren N., Allan I.J., Carter J.E., House W.A., Parker A. (2003). Pesticides and other micro-organic contaminants in freshwater sedimentary environments—A review. Appl. Geochem..

[36] MFDS Common Guidelines for Risk Assessment of Human Applications. https://www.mfds.go.kr.

[37] CODEX Alimentarius Commission (2009). Guidelines for the Design and Implementation of National Regulatory Food Safety Assurance Programme Associated with the Use of Veterinary Drugs in Food Producing Animals. CAC/GL.

[38] KNHANES (Korea National Health and Nutrition Examination Survey) (2021). https://knhanes.kdca.go.kr/knhanes/main.do.

