# Supplementary material for: Residue Monitoring and Risk Assessment of 51 Pesticides in Domestic Shellfish and Seaweed Using GC-MS/MS
Source: Int J Mol Sci. 2025 May 16;26(10):4765. doi: 10.3390/ijms26104765 (PMC12112213; doi:10.3390/ijms26104765)
Supplement: Supplementary file 1 [file ijms-26-04765-s001.zip › ijms-3623386-supplementary.pdf]

Supplementary Material

Table S1. Experimental conditions for GC-MS/MS in the multiple reaction monitoring mode

Pesticide	Retention time (min)	Precursor ion (m/z)	Product ion (m/z)	Collision Energy (eV)
2,4′-DDD	11.54	237	165	35
		235	165	35
2,4′-DDE	11.00	248	176	45
		246	176	45
2,4′-DDT	11.54	237	165	35
		235	165	35
4,4′-DDD	11.54	237	165	35
		235	165	30
4,4′-DDE	11.00	248	176	45
		246	176	45
4,4′-DDT	13.12	237	165	35
		235	165	35
Acetochlor	9.55	223	132	35
		146	130	35
Alachlor	9.68	237	160	10
		188	160	10
Aldrin	10.18	263	193	45
		263	191	45
Ametryn	9.66	227	185	5
		227	170	15
Atrazine	8.62	215	200	10
		215	58	15
α-BHC	8.40	217	181	10
		181	145	20
β-BHC	8.40	217	181	10
		181	145	20
γ-BHC	8.40	217	181	10
		181	145	20
Boscalid	16.57	140	112	15
		140	76	35
Buprofezin	11.53	175	132	15
		172	57	15
Carfentrazone-ethyl	12.47	340	312	15
		312	151	30
α-Chlordane	11.17	375	266	35
		373	266	30
β-Chlordane	10.97	375	266	35
		373	266	35

Chlorothalonil	9.17	266	231	25
		266	170	35
Chlorpyrifos	10.19	314	258	25
		199	171	20
Cypermethrin	16.59	165	91	15
		163	127	5
Deltamethrin	18.10	253	174	10
		253	93	25
Dieldrin	11.50	265	193	45
		263	193	45
Dimethametryn	10.56	212	94	30
		212	68	45
Diphenylamine	7.82	169	168	20
		168	167	30
Endosulfan sulfate	12.17	272	237	20
		270	235	20
		239	204	20
α-Endosulfan	11.14	241	206	20
		205	170	20
β-Endosulfan	11.97	207	172	20
		205	170	20
Endrin	11.50	263	193	45
		263	191	45
Fenitrothion	9.93	277	260	5
		277	109	20
Fipronil	10.68	367	255	15
		367	213	35
Heptachlor	9.74	274	237	20
		272	237	20
Heptachlor epoxide (cis)	10.73	353	253	20
		217	182	30
Heptachlor epoxide (trans)	10.73	353	253	25
		217	182	30
Hexachlorobenzene	8.51	284	249	25
		284	214	45
Iprobenfos	9.25	204	121	45
		204	91	10
Isoprothiolane	11.30	231	189	10
		189	89	25
Mirex	14.93	272	237	20
		272	143	50
		270	235	20
Nonachlor (cis)	11.23	409	300	25
		407	109	25
Nonachlor (trans)	11.23	407	300	30
		407	263	30
Oxadiazon	11.41	258	175	10

		175	112	20
Pendimethalin	10.60	194	208	5
		252	162	10
Permethrin	15.74	183	168	20
		183	155	10
Prometryn	9.70	241	199	5
		241	184	15
Tebuconazole	12.97	252	127	30
		250	125	30
Terbutryn	9.87	241	185	5
		185	170	10
Tetraconazole	10.27	336	218	25
		336	204	40
Thifluzamide	11.86	194	166	15
		194	125	35
Trifluralin	8.33	306	264	10
		264	206	5
